# USA: Ophthalmologic Evaluation and Management of Acute Stevens-Johnson Syndrome

**DOI:** 10.3389/fmed.2021.670643

**Published:** 2021-07-07

**Authors:** Darren G. Gregory

**Affiliations:** Sue Anschutz-Rodgers Eye Center, University of Colorado, Aurora, CO, United States

**Keywords:** Stevens-Johnson syndrome, toxic epidermal necrolysis, amniotic membrane, ocular surface, USA

## Abstract

Stevens-Johnson syndrome (SJS) and toxic epidermal necrolysis (TEN) can cause significant damage to the ocular surface and eyelids. The sloughing and inflammation of the ocular mucosal epithelium during the acute phase may lead to scarring sequelae of the eyelids and ocular surface, resulting in pain and vision loss. Amniotic membrane transplantation (AMT) to the eyes and eyelids during the initial 1–2 weeks of the disease can decrease the chronic sequelae. The main development in the ophthalmologic treatment of SJS/TEN in the USA over the last 15 years has been the use of AMT on the ocular surface and eyelids during the acute phase. The evolution of AMT techniques, refinement of the evaluation of the eyes in acute SJS, and the efforts to increase the use of AMT in the USA are discussed.

## Introduction

Stevens-Johnson syndrome (SJS) and its more severe variant, toxic epidermal necrolysis (TEN), are rare diseases that cause acute blistering of the skin and mucous membranes. Both diseases are most commonly drug-induced and the effects on the ocular surface can be devastating, potentially yielding severe scarring, and dry eye problems, as well as debilitating photophobia and decreased vision. The acute phase of the illness can vary in length, but essentially corresponds to the period of active blistering and epithelial sloughing (1). This period can range from a few days in mild cases up to a few weeks in severe cases. In 2002 the first case report was published describing the use of amniotic membrane transplantation (AMT) to treat the ocular surface inflammation in acute SJS (2). Since then numerous other investigators have shown that when it is applied to the ocular surface during the early portion of the acute phase of the illness, AMT can minimize the damage that leads to the late-phase chronic cicatricial problems.

At the University of Colorado we began using AMT to treat acute SJS in 2005 and soon realized the benefits it provided. In 2008 we published a “how to” guide for the ophthalmologic management of acute SJS (3). Many of our chronic SJS patients used customized scleral contact lenses (PROSE devices, Boston Foundation for Sight, Needham, Mass) as a treatment for their severe dry eye problems. Through the Boston Foundation for Sight and the network of ophthalmologists who used their devices, an interest group of ophthalmologists who care for acute SJS patients developed. All were at large ophthalmology training centers with an affiliated burn intensive care unit (ICU). The bulk of the publications and advances in the care of acute SJS patients in the USA have come from the efforts of this group. Principal members are from the University of Colorado, The Massachusetts Eye and Ear Infirmary/Harvard University in Boston, Loyola University Medical Center/Loyola University of Chicago, the University of Miami, Weill-Cornell Medical Center in New York City, and Brooke Army Medical Center in San Antonio. This group, along with numerous international experts, published a consensus paper in 2016 regarding the ophthalmologic evaluation and management of SJS/TEN (1). The current article will review the rationales for the current evaluation and management of the acute ocular manifestations of SJS and TEN in the USA.

## Evaluation

With an incidence of just over 12 cases per million persons per year, SJS/TEN seems to be more common in the USA than older reports have suggested (4). The eyes are affected in a majority of SJS/TEN patients (5, 6). The epithelial sloughing and inflammation that is characteristic of the disease can involve the eyelid margins and eyelashes, the palpebral and bulbar conjunctiva, and the corneas. Even in cases where the extent of the skin sloughing on the rest of the body is limited, the extent of the ocular surface epithelial sloughing can be significant (7, 8). It is therefore important that all acute SJS/TEN patients have an urgent evaluation of the eyes by an ophthalmologist, regardless of the severity of the overall skin involvement. Damage to the ocular surface begins with widespread necrosis in the deep epidermal layers and the intense inflammation that follows. Areas that experience extensive epithelial necrosis will tend to heal in with scar tissue rather than normal mucosa. The mucosal damage that begins in the first few weeks of the illness leads to the relentless cascade of challenging ocular surface problems that characterize the chronic phase of the disease months and years after the acute episode. The extent of the epithelial sloughing of the conjunctiva and cornea (delineated by fluorescein staining) is the key exam finding that determines the severity of the acute eye involvement and helps guide management (9). Inspection of the fornices is crucial, as there may be extensive sloughing of the palpebral conjunctiva even in cases where there is relatively mild bulbar conjunctival sloughing. Simply sweeping for symblephara is not sufficient. Controlling the intense inflammation that is leading to the symblepharon formation should be the priority.

**Figure 1:**
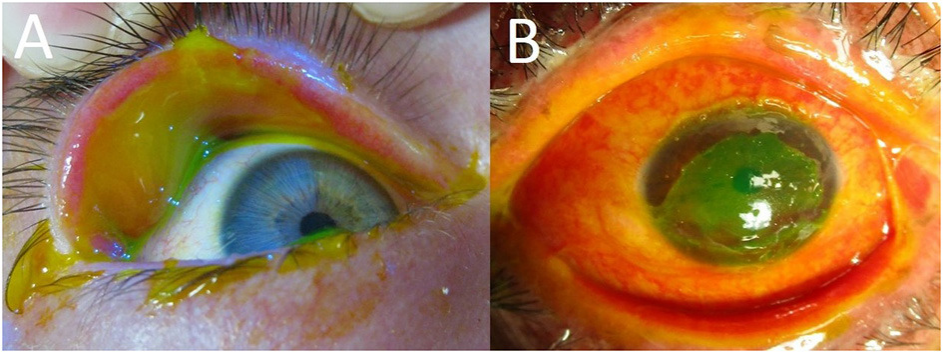
**(A)** Fluorescein staining in an acute SJS patient showing extensive palpebral conjunctival sloughing despite limited bulbar conjunctival sloughing. **(B)** Severe eye involvement in an acute SJS patient (extensive sloughing of the corneal epithelium, bulbar conjunctiva, palpebral conjunctiva, and lid margins).

In the acute phase, mild eye involvement is characterized by a conjunctivitis that may be accompanied by small, well-demarcated conjunctival epithelial defects that are easily appreciated with fluorescein staining done at the bedside. In seemingly mild cases it is still important to inspect the fornices and palpebral conjunctiva for sloughing that would not otherwise be seen (Figure 1A). Inspection and saline rinsing of the ocular surface (including the fornices) should be performed daily at least until there are signs of improvement. Enlargement of epithelial defects, corneal epithelial sloughing, or the formation of symblephara indicate worsening that should prompt consideration of more significant ophthalmologic treatments, such as AMT.

Severe cases have more diffuse, destructive conjunctival inflammation with pseudomembranous and membranous conjunctivitis. Fluorescein staining is extensive, involving much of the ocular surface (Figure 1B). These raw surfaces can lead to adhesion formation between the palpebral and bulbar conjunctiva. Although this symblepharon formation is concerning, the larger problem is the underlying intense inflammation that is leading to the symblepharon in the first place. This inflammation can damage both goblet cells and accessory lacrimal glands on the ocular surface, as well as the secretory ductules of the main lacrimal gland (10). In such cases, urgent AMT is indicated to suppress the destructive inflammation and to limit the formation of cicatrized epithelium.

Damage to the eyelids in the acute phase can also be a significant source of longer term visual morbidity. Inflammation of the lid margins can cause occlusion of meibomian gland orifices and damage to the meibomian glands (11, 12). Cicatricial changes may alter eyelid and eyelash anatomy, resulting in entropion, trichiasis, and distichiasis. The abnormal eyelashes can rub the already compromised ocular surface and cause irritation, corneal abrasions, corneal ulceration, and corneal scarring. Keratinization of the lid margins and palpebral conjunctiva can also add to the discomfort and corneal damage in the chronic phase of the disease, so sloughing of the palpebral conjunctiva and lid margin ulceration in the acute phase is particularly concerning (13).

Severe cases may also yield limbal stem cell failure. There is probably some direct injury to the stem cells during the acute phase combined with ongoing damage from the abnormal tear film and mucosal surfaces in the years following the acute illness. The limbal stem cell damage can lead to opacification and “conjunctivalization” of the cornea, decreased vision, and chronic pain and photophobia. All of these ocular surface abnormalities make the prognosis for corneal transplantation poor. Prevention of the damage in the acute phase of the illness can greatly decrease the risk for these long-term, debilitating sequelae (14, 15). Early AMT (ideally in the first week of the illness) has been shown to be an effective means of suppressing the inflammation of the ocular mucosal membranes and minimizing the acute phase damage to these tissues (1).

## Management

### Medical Management

The extensive loss of skin and mucous membranes in SJS/TEN puts patients at risk for sepsis and pneumonia, with mortality rates approaching 40% in some series (16). Early referral to a facility experienced in the care of burn patients significantly decreases mortality rates (17–19). In the USA, this is commonly a regional burn ICU. The life-threatening nature of the disease, however, can initially cause the eye involvement to be overlooked or deprioritized, even at facilities specialized in the care of burn patients. A 2015 survey of North American burn centers found that only 66% routinely include ophthalmology consultation as part of the initial evaluation and care of SJS/TEN patients (20). It is crucial for ophthalmologists to help establish evaluation and treatment protocols in burn centers so that the urgent ophthalmologic evaluation of all SJS/TEN patients becomes the standard practice. Additionally the consulting ophthalmologist must be well-versed in the current evaluation and treatment recommendations for the SJS/TEN. Patients with mild skin involvement may still have significant mucosal inflammation and need urgent ophthalmologic evaluation during the acute phase. The clinical severity can progress quickly in the first few days of the illness, so daily eye exams are needed until it is clear that the mucosal inflammation is subsiding. Failure to appreciate the severity of eye involvement in patients with less severe skin involvement can lead to delays in transfer to a facility experienced in the care of SJS patients. Such delays can potentially cause worse visual outcomes for the patients. As part of an overall effort to improve awareness of AMT's benefits in acute SJS, Loyola University Medical Center convened a free, virtual national educational symposium specifically for burn centers in March 2021. Continued collaboration with burn centers is planned.

The ophthalmologic management of acute SJS/TEN should focus on infection prophylaxis, symblepharon prevention, and minimization of destructive inflammation. In the USA, systemic treatments are generally directed by the burn surgeons and medical specialists in charge of the overall care of the patient. Unfortunately there are few studies that examine the effects of systemic acute phase treatments on the eyes. In 2009 it was reported that high dose systemic and topical corticosteroids within the first 4 days after the onset of illness could decrease the severity of the ocular surface damage (21). The use of systemic corticosteroids in the acute phase has historically been controversial due to concerns over possible increased mortality (22, 23). Multiple reports suggest that with close monitoring, however, intensive topical corticosteroid drops may be safely used to help decrease the ocular surface damage during the acute phase (21, 24). Subconjunctival triamcinolone injections have also been utilized in combination with AMT (25). Although they may play a beneficial role in decreasing the ocular surface inflammation in acute SJS, topical steroids alone are not sufficient treatment in severe cases.

Studies of other systemic treatments have mostly looked at mortality as the main outcome measure. Other than possibly corticosteroids, no other systemic treatments have been shown to have a benefit for the ocular surface (26, 27). This may be more due to a lack of studies specifically investigating this, rather than studies showing an actual lack of benefit. In a recent review of studies examining the use of systemic cyclosporine, however, no statistically significant benefit for the eyes was found (28).

Milder cases with non-membranous conjunctivitis and no lid margin or corneal involvement may be managed with daily exams, topical antibiotics, and topical steroid medications. Mild cases have a low risk for developing cicatricial sequelae and many will not progress to more severe involvement. Close monitoring is still required, however, until it is clear that no worsening is occurring. If membranous conjunctivitis, mucosal adhesions, or lid margin sloughing do develop, then urgent AMT should be considered.

### Surgical Management

Cryopreserved AMT to the ocular surfaces during the acute phase of SJS and TEN has been described by multiple groups (1, 24, 29–33). The use of AMT in this setting has also been summarized in multiple reviews (2, 3). Additionally, a randomized control trial has established that AMT is more effective than medical management in the prevention of longer term cicatricial sequelae (34). The evidence supporting the early use of AMT in SJS/TEN cases with severe ocular involvement is increasingly strong. AMT seems to be most beneficial if applied during the first week of illness, the earlier the better (9). After the first 2weeks the process of scarring becomes more prominent in severe cases. Minimizing the inflammation and mucosal damage in the early phases of the disease decreases the severity of this scarring. The amniotic membrane (AM) degrades within 7–10 days following application, however, and may need to be reapplied in more severe cases with persistent mucosal sloughing.

Numerous techniques have been described for the application of AMT. It can be performed under local anesthesia at the bedside or under general anesthesia in an operating room. Regardless of the exact technique, however, the AMT should ideally cover the lid margins, palpebral conjunctiva and ocular surface, including the cornea. The bedside methods use a single large sheet of cryopreserved AM which is initially positioned over the eyelids and eye. It is then sutured (35) or glued (36) to the eyelid skin of the lower lid then folded up and over the lid margin into the fornix where it is held in place by a custom-made, ring-shaped conformer constructed from pliable, small-bore intravenous tubing. The superior portion of the AM sheet covering the globe is then reflected out of the superior fornix and over the upper eyelid margin and then fixated to the skin just beyond the lid margin and lashes using glue or sutures. The main advantage of these techniques is the logistical simplicity, allowing effective AM coverage of the lid margins and all conjunctival surfaces without the need for an operating microscope or a trip to the operating room. Transporting these critically ill patients to an operating room may be challenging or even impossible, so having bedside options is beneficial. Additionally, increasing the simplicity and ease of AMT may allow more widespread adoption of the procedure.

**Figure 2:**
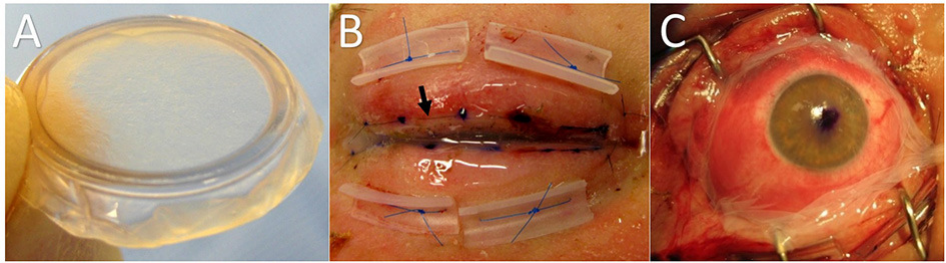
**(A)** Prokera self-retaining amniotic membrane. Useful only when bulbar conjunctival sloughing is limited. Amniotic membrane grafting to the lid margins and palpebral conjunctiva is still necessary even when a Prokera has been used on the surface of the globe. **(B)** Lid margin and palpebral conjunctival amniotic membrane. The black arrow shows the running 8–0 nylon suture 1–2 mm peripheral to the lid margin. Once fixated to the external lid skin by the 8–0 nylon, the membrane is reflected over the lid margin and onto the palpebral conjunctiva where it is fixated with a pair of double-armed 6-0 polypropylene sutures. Both needles of each suture are passed full-thickness through the membrane and the eyelid and then tied externally over bolsters. **(C)** SJS patient with significant bulbar conjunctival sloughing treated with a sheet of cryopreserved amniotic membrane rather than a Prokera. The running perilimbal 10-0 nylon suture and oblique quadrant interrupted sutures have not yet been placed.

Whenever possible, at our facility we still prefer to perform AMT under an operating microscope in an operating room. We use one half of a 3.5 cm^2^ sheet of cryopreserved AM (Amniograft, Bio-Tissue, Miami, FL) on each eyelid and a full 3.5 cm^2^ AM sheet or a Prokera (Bio-Tissue, Miami, FL) on the ocular surface. A Prokera is a 16 mm diameter thin plastic ring with a sheet of AM stretched across the lumen of the ring (Figure 2A). It is placed over the cornea like a contact lens. The amniotic membrane portion only covers the cornea and perilimbal conjunctiva, however, so it should only be used in cases that have limited bulbar conjunctival sloughing.

For the eyelid treatment, the eyelashes are trimmed close to the skin. Then a long edge of the AM sheet is placed along the eyelid margin with the stromal surface against the skin. An 8-0 nylon running suture fixates the membrane edge along the eyelid skin ~2 mm from the lid margin. Then the rest of the AM sheet is reflected over the lid margin and into the fornix. A muscle hook is helpful for spreading the membrane out and keeping it in the proper position along the back of the eyelid while further sutures are being placed. Both ends of a double-armed 6-0 polypropylene suture are passed through the AM as deep in the fornix as possible and then full-thickness through the eyelid to be tied over a bolster on the external eyelid skin surface. Two such sutures and bolsters are used on each eyelid to fixate the sheet of AM to the palpebral conjunctiva (Figure 2B).

Following the application of AM to the lid margin and palpebral conjunctiva of each eyelid, we then simply place a Prokera on the ocular surface in most cases. The Prokera can be used in place of sutured AMT for the ocular surface, but only in cases with very limited sloughing of the bulbar conjunctiva. If there is extensive bulbar conjunctival sloughing we recommend suturing a sheet of AM to surface of the globe instead of placing a Prokera since a Prokera provides incomplete coverage of the bulbar conjunctiva. When complete coverage of the bulbar conjunctiva is needed, a full 3.5 cm^2^ piece is centered over the cornea and sutured to the conjunctiva using a 10-0 nylon suture running circumferentially around the cornea 1–2 mm posterior to the limbus. An ink mark is placed on the center point of the AM sheet to help keep it properly centered during positioning and suturing (Figure 2C). Epinephrine drops (1:1,000) applied to the ocular surface prior to suturing help to decrease bleeding. After the perilimbal running suture is completed, single interrupted 10-0 nylon sutures are placed in each oblique quadrant and at the medial and lateral canthi. The suture tails are left long so they will lay flat on the ocular surface. A symblepharon ring is then placed on the eye to help maintain the fornices and to optimize the apposition of the amniotic membrane to the mucosal surfaces.

### Postoperative Care

Postoperative care starts with showing the nursing staff how to properly apply the eye medications, particularly the eyelid ointment. Combination tobramycin/dexamethasone ointment is applied to the eyelid margins and eyelashes 4 times per day to minimize inflammation and to prevent desiccation of the lid margin amniotic membrane. The importance of this needs to be stressed to the nurses caring for the patient. An ophthalmologic exam is performed daily and includes rinsing the eyes with sterile saline to remove the buildup of ointment and serosanguinous debris, which can be significant. The corneas are evaluated for the presence of infiltrates under the AM. Topical quinolone drops and corticosteroid drops are applied 4 times per day. The amniotic membranes degrade after about a week in most cases and should be reapplied if there are areas of persistent, severe inflammation.

## Discussion

SJS and TEN are among the worst diseases of the ocular surface and their incidence is likely higher than previously reports have suggested. The long-term cicatricial sequelae of the diseases can be devastating to patients, and efforts to correct these chronic problems are prone to failure. Applying cryopreserved amniotic membrane to the eyes and eyelids during the acute phase of the disease greatly decreases the occurrence of severe cicatricial sequelae and the resultant visual problems. Early intervention with AMT during the acute phase is crucial in severe cases because the window of opportunity is short and the potential consequences of the disease are significant. Although early AMT is the recommended treatment for severe cases, the management of SJS in the United States remains quite variable. Severe cases are often transferred to intensive care units specializing in the care of burn patients, but not always. The decisions on the systemic management of the disease are made by burn surgeons and other medical specialists. Ophthalmologists may consult on the specific treatment of the eyes, but generally play no role in the systemic management. There is still no national consensus on what systemic anti-inflammatory agents, if any, should be used in the acute phase of the disease.

Although the ophthalmologic use of AMT in the acute phase appears to be slowly increasing, delays in the referral of patients to tertiary care burn centers where AMT can be performed still occur. Additionally, consulting ophthalmologists outside of large teaching centers may only rarely see cases of acute SJS and, thus, may not be aware that AMT can be an effective treatment in the acute phase. Not all ophthalmologists use AM on a regular basis and most facilities do not keep the necessary AM in stock. Getting the necessary AM delivered presents both a hurdle and a delay for treatment. As a result, AMT remains underutilized in acute SJS.

Increasing the awareness and utilization of AMT as a treatment option remains a top priority for American ophthalmologists who regularly care for patients with acute SJS and TEN. Delivering lectures to burn centers and publishing guidelines for the eye care of acute SJS in burn journals as well as ophthalmology journals are key steps in the awareness campaign. Additionally, educating cornea surgical trainees on the use of AMT in acute SJS is increasing awareness each year. Unfortunately there is still no national database to monitor treatment and outcomes, nor is there a national treatment guideline that is adhered to. As a result the ophthalmologic management of acute SJS remains quite variable throughout the United States. Urgent AMT is commonly performed at large academic institutions with affiliated burn centers, but is less widely used outside of those settings.

## Data Availability Statement

The original contributions presented in the study are included in the article/supplementary material, further inquiries can be directed to the corresponding author/s.

## Author Contributions

The author confirms being the sole contributor of this work and has approved it for publication.

## Conflict of Interest

The author declares that the research was conducted in the absence of any commercial or financial relationships that could be construed as a potential conflict of interest.
